# LATS1/2 kinases trigger self-renewal of cancer stem cells in aggressive oral cancer

**DOI:** 10.18632/oncotarget.26583

**Published:** 2019-02-01

**Authors:** Masami Nozaki, Norikazu Yabuta, Moe Fukuzawa, Satomi Mukai, Ayumi Okamoto, Towa Sasakura, Kohshiro Fukushima, Yoko Naito, Gregory D. Longmore, Hiroshi Nojima

**Affiliations:** ^1^ Department of Cell Biology, Research Institute for Microbial Diseases, Osaka University, Suita, Osaka 565-0871, Japan; ^2^ Department of Molecular Genetics, Research Institute for Microbial Diseases, Osaka University, Suita, Osaka 565-0871, Japan; ^3^ ICCE Institute, Washington University, St Louis, MO 63110, USA; ^4^ Department of Oncogene Research, Research Institute for Microbial Diseases, Osaka University, Suita, Osaka 565-0871, Japan; ^5^ Division of Cancer Biology, Aichi Cancer Center Research Institute, Chikusa-ku, Nagoya City, Aichi 464-8681, Japan; ^6^ Division of Cancer Cell Regulation, Aichi Cancer Center Research Institute, Chikusa-ku, Nagoya City, Aichi 464-8681, Japan

**Keywords:** Hippo pathway, LATS, SNAIL, oral cancer, cancer stem cells (CSCs)

## Abstract

Cancer stem cells (CSCs), which play important roles in tumor initiation and progression, are resistant to many types of therapies. However, the regulatory mechanisms underlying CSC-specific properties, including self-renewal, are poorly understood. Here, we found that LATS1/2, the core Hippo pathway-kinases, were highly expressed in the oral squamous cell carcinoma line SAS, which exhibits high capacity of CSCs, and that depletion of these kinases prevented SAS cells from forming spheres under serum-free conditions. Detailed examination of the expression and activation of LATS kinases and related proteins over a time course of sphere formation revealed that LATS1/2 were more highly expressed and markedly activated before initiation of self-renewal. Moreover, TAZ, SNAIL, CHK1/2, and Aurora-A were expressed in hierarchical, oscillating patterns during sphere formation, suggesting that the process consists of four sequential steps. Our results indicate that LATS1/2 trigger self-renewal of CSCs by regulating the Hippo pathway, the EMT, and cell division.

## INTRODUCTION

Cancer stem cells (CSCs), also called tumor-initiating cells (TICs), drive tumorigenesis and tumor heterogeneity through their abilities to self-renew and generate tumorigenic progeny. These cells are also resistant to a wide range of therapeutic agents, including chemotherapy and radiotherapy, and thus promote cancer progression and tumor recurrence [1, 2, 3, 4, 5]. Importantly, CSC-specific properties such as self-renewal and chemoresistance are associated with the epithelial–mesenchymal transition (EMT), a cellular program that promotes invasion and metastasis during cancer development [6, 7, 8, 9, 10, 11]. For instance, EMT induced by ectopic expression of EMT-regulated transcription factors (EMT-TFs) such as SNAIL (*SNAI1*) and TWIST endows mammary epithelial cells with CSC properties [6]. In addition, a study using a knock-in reporter mouse line for *Snail* (Snail-YFP) demonstrated that breast TICs expressing Snail undergo the EMT [12]. These findings imply that, through activation of EMT-TFs, especially SNAIL, the EMT is a leading cause of cancer stemness in a variety of tumors [13, 14, 15]. Moreover, diverse signaling pathways, including Hippo, WNT, SHH (sonic hedgehog), NOTCH, and the DNA damage response (DDR), are involved in CSC properties and the EMT [16, 17, 18, 19, 20, 21]. Although these studies have advanced our understanding, the molecular mechanisms underlying CSC-specific properties, especially their capacity to initiate and maintain self-renewal, have yet to be fully elucidated.

LATS1 and LATS2 (LATS1/2), the core kinases of the Hippo pathway, regulate tissue homeostasis and tumorigenesis by preventing cell proliferation or promoting cell death through a phosphorylation signaling cascade [22, 23, 24]. In this cascade, LATS1/2 are activated by two upstream kinases, MST1 and MST2, in response to divergent stimuli such as cell–cell contact, serum starvation, cell polarity, and mechanical features, and then directly phosphorylate two transcriptional co-factors, YAP (on S127) and TAZ (on S89). Phosphorylation represses the nuclear activities of YAP/TAZ by promoting their association with 14-3-3 protein, resulting in their cytoplasmic retention. LATS1/2 also promote the degradation of YAP/TAZ proteins by phosphorylation-mediated ubiquitination via an interaction with the β-TrCP E3 ubiquitin-ligase complex. Consistent with this, in many human malignant tumors, such as liver, colon, breast, and oral cancers, YAP/TAZ are activated, whereas LATS1/2 are inactivated [25, 26, 27, 28]. Notably, LATS1/2 play pivotal roles in the control of cell fate, not only by inhibiting YAP/TAZ in a manner dependent on the canonical Hippo pathway, but also by regulating a tumor-suppressive transcriptional factor p53, Polycomb repressive complex 2 (PRC2), SNAIL, and cell cycle checkpoint regulators including mitotic kinases of the Aurora family, the cofilin regulator LIM-kinase 1, and the centrosomal protein phosphatase CDC25B [29, 30]. Thus, LATS1/2 also regulate chromosomal instability, DDR, EMT, metastasis, cell division, and cell stemness. Recent studies showed that YAP/TAZ are required for the maintenance and expansion of CSCs in various solid tumors [28, 31]. For instance, TAZ confers self-renewal capacity, a CSC property, on breast, brain, and oral cancer cells, probably by inducing the EMT [21, 32, 33, 34]. Similarly, YAP confers some CSC properties, such as sphere formation and chemoresistance, on hepatocellular carcinoma, esophageal cancer, osteosarcoma, and basal-like breast cancer cells by coordinating the expression of interleukin 6 (IL-6) and stemness marker proteins such as SOX2, SOX9, and CD90 [35, 36, 37, 38]. Nevertheless, the biological roles of LATS1/2, as well as the mechanisms by which they enable cancer cells to acquire and maintain CSC properties, are incompletely understood.

The most frequently observed form of head-and-neck cancer in Southeast Asia is oral squamous cell carcinoma (OSCC), which is the most commonly emerging cancer worldwide. Survival rates of patients with advanced OSCC have not increased significantly in recent years [39]. This is partly due to the large proportion of patients with advanced stages of disease, which may not respond to any available therapies [40, 41]. To develop effective therapeutic strategies against OSCC, it is crucial to understand the detailed molecular mechanisms underlying CSC properties in this disease. Such knowledge would facilitate the identification of useful CSC markers [42]. Successful isolation of CSCs from OSCCs (e.g., the SAS cell line) using non-adhesive culture systems represents a promising advance in this research field. SAS cells exhibit the full spectrum of CSC-specific properties: stemness, self-renewal, chemo- and radioresistance *in vitro*, and tumor initiation capacity *in vivo* [43].

In this study, using SAS cells as a model of CSCs in OSCC, we showed that LATS1/2 are essential for self-renewal of CSCs, and in particular for the initiation of sphere formation. Notably, we found that the expression patterns of LATS1/2 oscillated over the course of sphere formation of CSCs under serum-free conditions, and that these kinases were activated just before self-renewal (cell division). This temporal pattern was associated with the hierarchical oscillating expression of TAZ (but not YAP), SNAIL, CHK1/2, and Aurora-A. Loss of any of the latter proteins prevented SAS cells from forming spheres. These results imply that the process of sphere formation in CSCs consists of four sequential steps. Based on these findings, we propose the existence of a special stage (the “pre-SR stage”) that serves as a preliminary step for the initiation of self-renewal.

## RESULTS

### LATS1 and LATS2 are overexpressed in SAS cells

SAS is an OSCC cell line that exhibits prominent CSC properties, including sphere formation, radioresistance, multidrug resistance, and tumor formation [43, 44, 45]. Spheres of SAS cells also express high levels of representative stem cell markers such as CD133 and ALDH1 and pluripotent transcription factors such as SOX2, OCT4, and NANOG. In contrast to SAS cells, OSCC cell lines such as HSC3 and HSC4 do not form spheres and are highly chemosensitive [44]. Thus, SAS cells are useful tools for analyzing the molecular mechanisms underlying CSC properties. Because SAS cells readily form spheres with CSC properties in non-adhesive culture systems [43, 45], they are an ideal model for investigating the self-renewal of CSCs.

**Figure 1:**
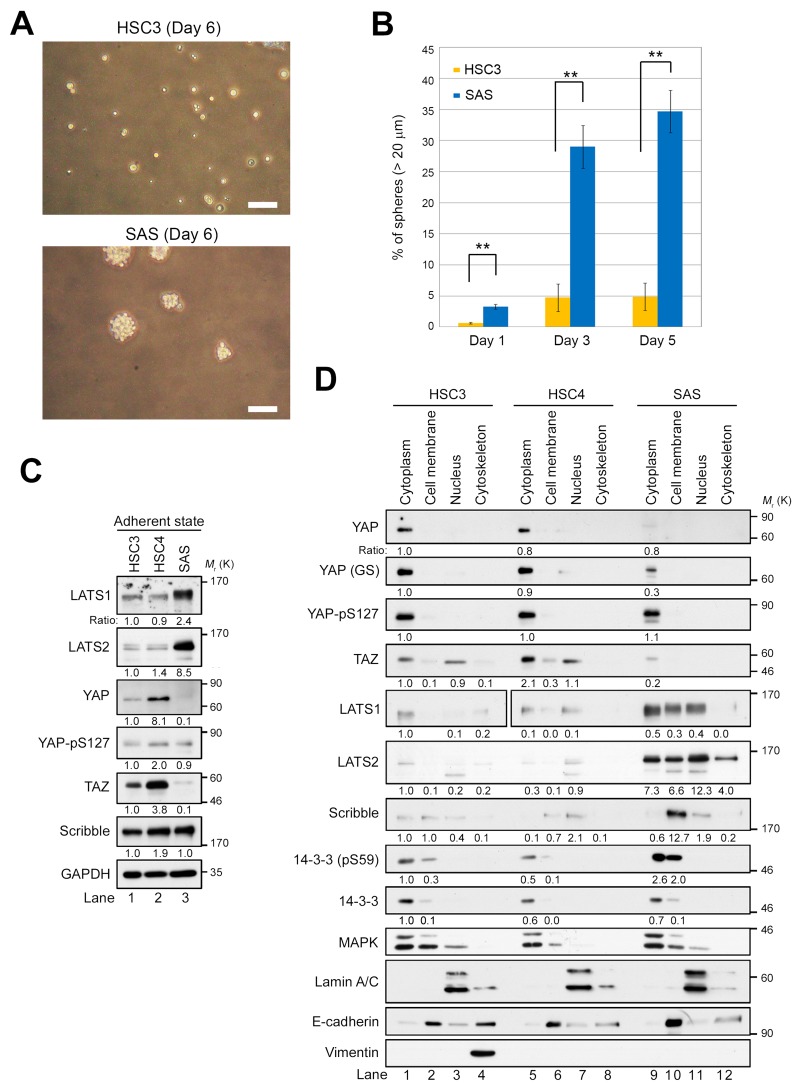
LATS1/2 are overexpressed and YAP/TAZ are downregulated in SAS cells in the adherent state **(A)** Phase contrast images of spheres from HSC3 (top) and SAS (bottom) cell lines seeded at 400 cells/cm^2^ in 100 mm low-adhesion sphere culture dishes and grown for 6 days. Scale bars represent 100 μm. **(B)** Quantification of sphere-forming ability of HSC3 (orange bars) and SAS (blue bars) cells. Bar graphs show the percentages of spheres (> 20 μm diameter) among total cells (HSC3, > 200 cells; SAS, > 170 cells). **(C)** Western blot analysis of the indicated Hippo pathway regulators in HSC3, HSC4, and SAS cells growing under adherent culture conditions. GAPDH was used as a loading control. The levels of the indicated proteins were normalized against the level of GAPDH. Expression patterns of other proteins, including α-tubulin, were shown in Supplementary Figure 1C. **(D)** HSC3, HSC4, and SAS cells were cultured under adherent conditions and fractionated into cytoplasm, cell membrane, nucleus, and cytoskeleton, followed by western blotting with the indicated antibodies. YAP and YAP (GS) are monoclonal (clone H-9) and polyclonal antibodies, respectively. 14-3-3 and MAPK are cytoplasmic markers; Lamin A/C is a nuclear marker; and E-cadherin is a cell membrane marker. Another cytoskeleton marker, vimentin, was detected only in the cytoskeleton fraction of HSC3, suggesting that HSC4 and SAS cells might retain epithelial properties.

To identify the signaling pathway driving cancer stemness (a cancer-initiating property) in OSCC, we examined self-renewal potential in SAS cells by assessing their capacity to form and propagate spheres *in vitro* (Figure 1A). When SAS and HSC3 cells were incubated on ultra–low attachment surface plates under sphere-forming conditions in the absence of serum, SAS cells formed spheres at high frequency, whereas HSC3 cells barely aggregated (Figure 1B). Consistent with previous results [44, 45], these observations indicate that SAS cells have significantly greater self-renewal potential than HSC3 cells.

Next, we asked whether the difference in CSC-related properties, including the ability to form spheres, between SAS, HSC3, and HSC4 cells is due to differential expression of the canonical Hippo pathway regulators such as LATS1/2 and YAP/TAZ. Under conventional adherent conditions in the presence of serum, protein levels of LATS1/2 were markedly higher in SAS cells than in HSC3 and HSC4 cells (Figure 1C). Consistent with a theory of the canonical Hippo pathway that LATS1/2 inhibit YAP and TAZ, YAP and TAZ were absent or present at very low levels in SAS cells (Figure 1C, lane 3). However, LATS1/2-mediated inhibitory phosphorylation of YAP-S127 (pS127) did not differ significantly among the three cell lines. Unexpectedly, HSC4 cells expressed higher levels of YAP and TAZ compared with HSC3 cells, independent of the levels of LATS1/2 (Figure 1C, lane 2). These results suggest that, in SAS cells, LATS1/2 are potentially overexpressed and probably activated, and thus inhibit YAP and TAZ without promoting their cytoplasmic retention. Indeed, western blot analysis of cell extracts fractionated into cytoplasm, cell membrane, nucleus, and cytoskeleton revealed that, in SAS cells, the level of YAP-pS127 was not elevated in the cytoplasm, even though LATS1/2 were highly expressed, and that the levels of YAP and TAZ were lower than in HSC3 and HSC4 (Figure 1D, lane 9). A portion of YAP and TAZ localized in the nuclear fraction in HSC3 and HSC4 cells, but not in SAS cells (Figure 1D, lanes 3, 7, and 11; Supplementary Figure 1B, lanes 3 and 7). Consistent with previous reports, LATS2 was present in all four fractions (Figure 1D, lanes 9–12). In addition, phosphorylation of 14-3-3 protein on S59, a target of LATS2 kinase [46], was elevated in the cytoplasm and cell membrane fractions of SAS cells, suggesting that LATS2 is activated in these cells.

When the Hippo pathway is activated, YAP and TAZ are destabilized by LATS1/2- and CK2-mediated phosphorylation, and subsequently degraded via the ubiquitin–proteasome system [47, 48]. To investigate the mechanism responsible for reduced expression of YAP in SAS cells, we treated cells with MG132, a proteasome inhibitor, and then fractionated extracts as in Figure 1D (Supplementary Figure 1A). MG132 treatment restored the YAP protein level (Supplementary Figure 1B, lane 9). We also found that Scribble, an upstream regulator of the Hippo pathway to determine the cell polarity, was overexpressed and localized to the cell membrane in SAS cells (Figure 1C, sixth panel from top; 1d, seventh panel from top). In epithelial breast cancer cells, Scribble facilitates interactions between the core Hippo kinases (MST1/2 and LATS1/2) and TAZ, which acts as a scaffold protein, thereby destabilizing and preventing activation of TAZ [21]. Accordingly, the downregulation and delocalization of Scribble may be sufficient for stabilization of TAZ in HSC3 and HSC4 cells. By contrast, in SAS cells, LATS1/2 might be activated, thereby downregulating TAZ by anchoring Scribble to the cell membrane. Indeed, in SAS cells LATS1/2 were activated by MST2 by phosphorylating their respective target sites, T1079 and T1041, (Supplementary Figure 1C, lane 3). Taken together with the phosphorylation of YAP-pS127 in SAS cells, these results suggest that YAP, and probably also TAZ, is inhibited in SAS cells via protein degradation, rather than via cytoplasmic retention mediated by binding to 14-3-3 protein.

### LATS1/2 are required for sphere formation in CSCs

**Figure 2:**
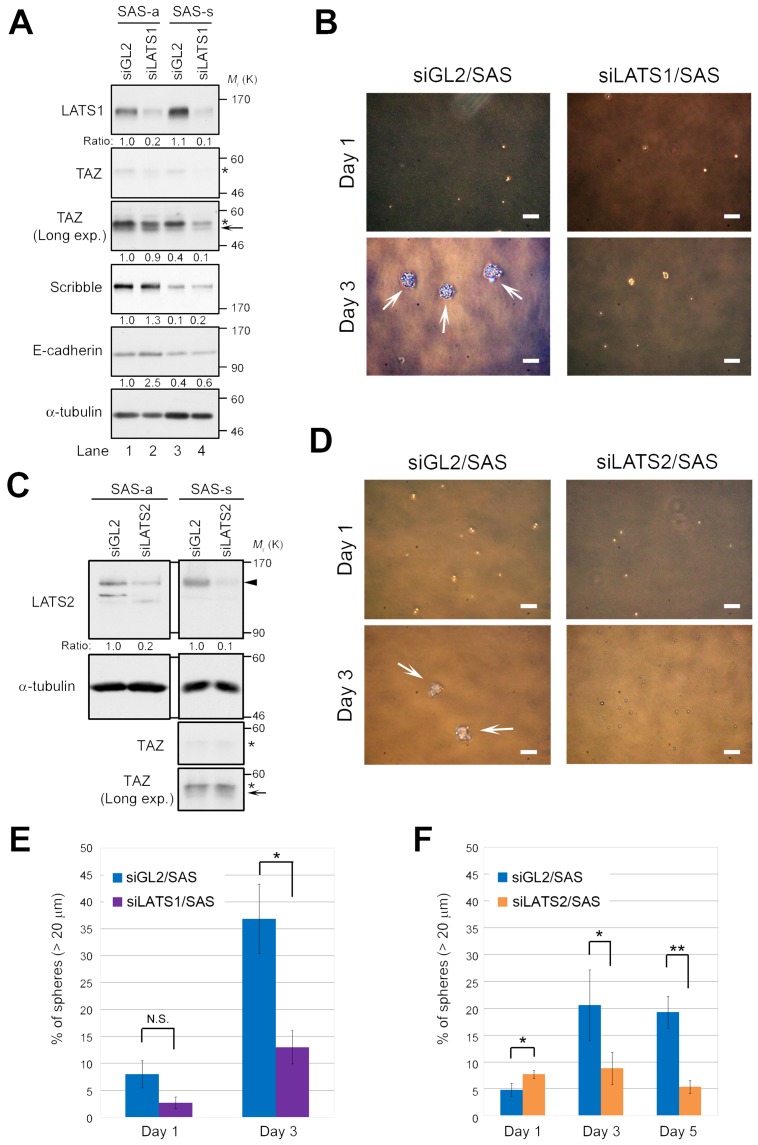
LATS1 and LATS2 are required for the sphere formation by SAS cells **(A)** SAS cells were transfected with siRNAs against LATS1 (siLATS1) or firefly luciferase as a negative control (siGL2), and cultured for 3 days under adherent culture conditions (SAS-a) or non-adherent sphere culture conditions (SAS-s). Cell lysates were subjected to western blotting with the indicated antibodies. A long-exposure western blot of TAZ (Long exp.) is shown in the third panel from the top. Asterisks and arrow show phosphorylated and non-phosphorylated (active) forms of TAZ, respectively. α-tubulin was used as a loading control. The levels of the indicated proteins were normalized against the level of α-tubulin. **(B)** Representative phase contrast images of LATS1-depleted (siLATS1/SAS) and control SAS (siGL2/SAS) cells, grown under sphere formation conditions, on days 1 and 3. White arrows indicate spheres. Scale bars represent 100 μm. **(C)** SAS cells were transfected with siRNAs against LATS2 (siLATS2) and cultured as in A. Cell lysates were subjected to western blotting with the indicated antibodies. Arrowhead indicates LATS2. Asterisks and arrow show phosphorylated and non-phosphorylated (active) forms of TAZ, respectively. α-tubulin was used as a loading control. The levels of LATS2 protein were normalized against the level of α-tubulin. **(D)** Representative phase contrast images of LATS2-depleted (siLATS2/SAS) and siGL2/SAS cells. Images were obtained as in B. White arrows indicate spheres. Scale bars represent 100 μm. **(E)** Frequency of sphere formation in siLATS1/SAS (purple bars) and siGL2/SAS (blue bars) cells, grown under sphere formation conditions, on days 1 and 3. More than 100 cells were counted on day 1, and more than 50 cells were counted on day 3. **(F)** Frequency of sphere formation in siLATS2/SAS (orange bars) and siGL2/SAS (blue bars) cells, grown under sphere formation conditions, on days 1, 3, and 5. More than 200 cells were counted.

To determine whether LATS1/2 are important factors for sphere formation in CSCs, we knocked down endogenous LATS1 or LATS2 using siRNA in SAS cells, and then grew the cells for one more day under conventional adherent culture conditions (termed “SAS-a” for “attached SAS”; Figure 2A, lanes 1 and 2) or in sphere-forming conditions without serum (termed “SAS-s” for “spheres”; Figure 2A, lanes 3 and 4). The level of LATS1 was elevated, whereas the level of Scribble was reduced, in SAS-s in comparison with SAS-a (Figure 2A, lanes 3 and 4). Expression of TAZ in SAS cells was very low (Figure 1C), and could not be detected unless the western blot exposure time was lengthened (Figure 2A, second and third panels from top); in any event, TAZ levels did not differ between the two conditions. Importantly, depletion of LATS1 inhibited sphere formation by SAS cells, whereas negative control cells (siGL2/SAS) formed spheres normally (Figure 2B). As with LATS1, knockdown of LATS2 also inhibited sphere formation by SAS cells (Figure 2C, 2D). Notably, depletion of LATS1 or LATS2 did not promote drastic accumulation of TAZ in SAS-a or SAS-s, although the active (non-phosphorylated) form of TAZ was slightly more abundant in LATS-knockdown cells (arrows in Figure 2A, 2C). To confirm the importance of LATS kinases in initiation of sphere formation, we measured the frequency of primary spheres > 20 μm in diameter in LATS1/2-depleted (siLATS1/SAS and siLATS2/SAS) and control SAS cells (siGL2/SAS). As expected, siLATS1/SAS and siLATS2/SAS suppressed sphere formation (frequency of spheres, ~5–10%), whereas siGL2/SAS generated a larger number of spheres after day 1 (20–40%) (Figure 2E, 2F). Double knockdown of LATS1/2 also suppressed the sphere formation (~15%), although the synergistic effect of LATS1 and LATS2 kinases was not observed in this assay (Supplementary Figure 1D, 1E). Furthermore, we confirmed that double knockdown of LATS1/2 suppressed sphere formation by another OSCC cell line, SCC-4 (Supplementary Figure 1F, 1G, 1H); however, unlike SAS cells, TAZ was detectably expressed in adherent SCC-4 cells (Supplementary Figure 1F, third panel from top). These results suggest that the LATS1/2 are required for self-renewal of CSCs, especially in the early initiation phase of sphere formation.

### Downregulation of TAZ is not essential for initiation of sphere formation in OSCCs

**Figure 3:**
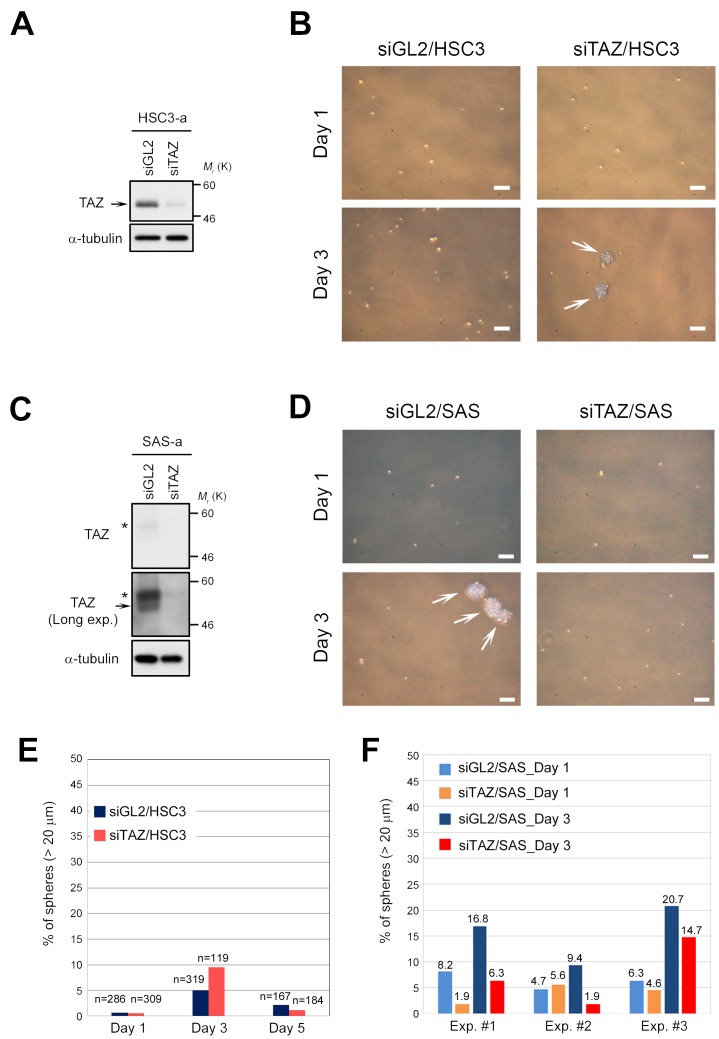
Downregulation of TAZ is insufficient to promote sphere formation in HSC3 cells **(A)** HSC3 cells were transfected with TAZ siRNA (siTAZ) and siGL2, and cultured for 3 days under adherent culture conditions (HSC3-a). Cell lysates were subjected to western blotting with the indicated antibodies. Arrow indicates TAZ. α-tubulin was used as a loading control. **(B)** Representative phase contrast images of TAZ-depleted (siTAZ/HSC3) and control HSC3 (siGL2/HSC3) cells, grown under sphere formation conditions, on days 1 and 3. White arrows indicate spheres. Scale bars represent 100 μm. **(C)** SAS cells were transfected with siTAZ and siGL2, and cultured as in A. Cell lysates were subjected to western blotting with the indicated antibodies. A long-exposure image of TAZ protein (Long exp.) is shown in the second panel; TAZ was expressed at a low level in SAS cells (see also Figure 1C). Asterisks and arrow show phosphorylated and non-phosphorylated (active) forms of TAZ, respectively. α-tubulin was used as a loading control. **(D)** Representative phase contrast images of TAZ-depleted (siTAZ/SAS) and siGL2/SAS cells. Images were obtained as in B. White arrows indicate spheres. Scale bars represent 100 μm. **(E)** Frequency of sphere formation in siTAZ/HSC3 (reddish orange bars) and siGL2/HSC3 (dark blue bars) cells, grown under sphere formation conditions, on days 1, 3, and 5. Numbers (n) above bar graphs represent total cell count, including spheres. **(F)** Frequency of sphere formation in siTAZ/SAS (orange and red bars) and siGL2/SAS (light blue and dark blue bars) cells, grown under sphere formation conditions, on days 1 and 3. More than 200 cells were counted on day 1, and more than 180 cells were counted on day 3. Data from three independent experiments (Exp. #1–#3) were shown by each bar graph. Numbers above bar graphs represent percentage of spheres.

In human oral cancer, TAZ promotes cancer stem cell maintenance [34]. We found that TAZ was downregulated in CSC-like OSCC cells, such as SAS, whereas its expression was maintained at a higher level in OSCC cells that do not form spheres, such as HSC3 and HSC4 (Figure 1B, 1C). To determine whether sphere formation by OSCC cells requires downregulation of TAZ, we transfected HSC3 cells with siRNA against TAZ (Figure 3A). Knockdown of endogenous TAZ weakly promoted the initiation of sphere formation until day 3 (< 10%), but this frequency of spheres was not maintained thereafter (Figure 3B, 3E). These results suggest that downregulation of TAZ does not confer high sphere formation capacity on HSC3 cells. Consistent with this, HSC4 cells, which also have low sphere formation capacity, also expressed higher levels of TAZ (Figure 1C, lane 2). Unsurprisingly, knockdown of TAZ did not increase sphere formation capacity in SAS cells (Figure 3C, 3D, 3F). Rather, knockdown of TAZ tended to decrease sphere formation capacity (red bars in Figure 3F). In contrast to the intracellular behavior of TAZ, a large fraction of YAP localized in the cytoplasm, but not the nucleus, in HSC3 cells (Figure 1D). Because YAP was also downregulated in SAS cells, we asked whether overexpression of YAP would prevent sphere formation in SAS cells (Supplementary Figure 2A). Overexpression of YAP did not prevent or promote sphere formation by SAS cells (Supplementary Figure 2B). Based on the consensus that cytoplasmic sequestration of YAP blocks its oncogenic activity, these results suggest that YAP makes a less important contribution than TAZ to growth and sphere formation of OSCC cells, including HSC3 and SAS.

### LATS1/2 are activated in CSCs at the preliminary stage of self-renewal

**Figure 4:**
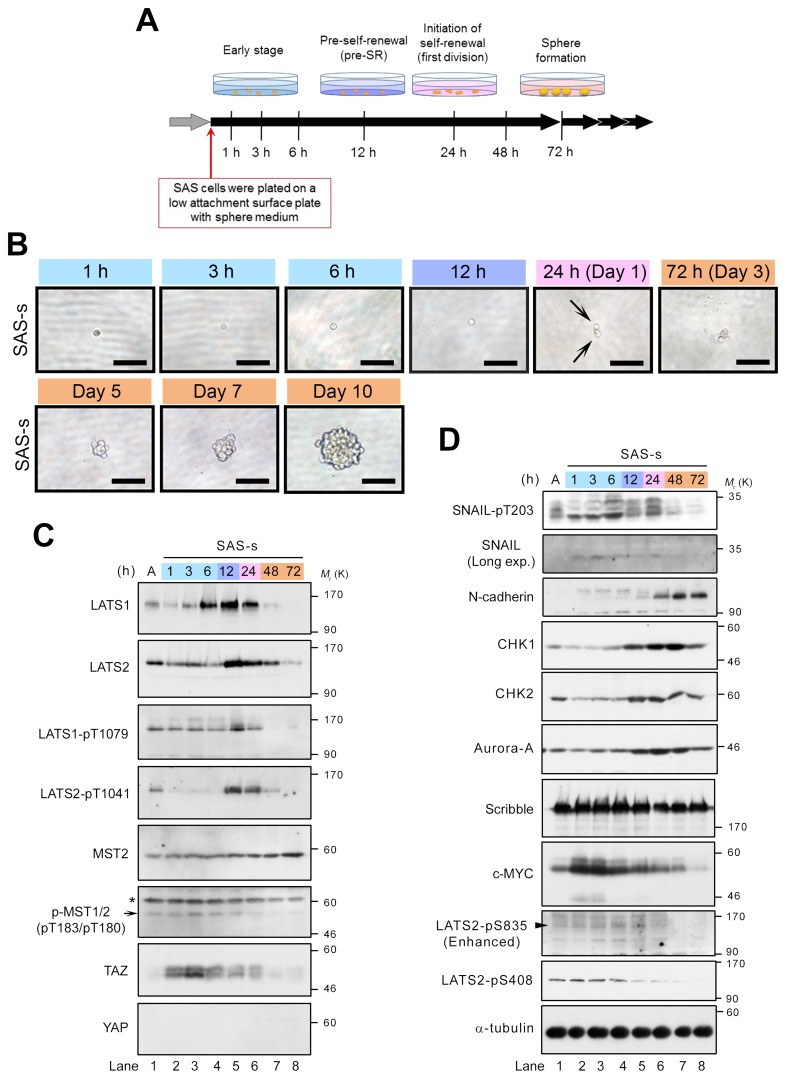
Expression and activation of LATS1 and LATS2 are elevated at the pre–self-renewal stage of sphere formation **(A)** Schematic depicting the time course analysis of sphere formation, which was classified into four stages: early stage (1–6 h after start of incubation in sphere medium), pre–self-renewal stage (pre-SR; at 12 h), self-renewal initiation stage (first division; at 24 h), and sphere formation stage (cell aggregation; from 48 h onward). **(B)** Representative bright field images of sphere-forming SAS cells during the time course analysis. SAS cells were plated on ultra–low attachment surface plates and cultured in sphere medium for the indicated times, as in A. The early, pre-SR, self-renewal initiation, and sphere formation stages are highlighted by labeling with light blue, purple, pink, and orange, respectively. Arrows indicate two daughter cells at first division (24 h after incubation in the sphere medium). Scale bars represent 100 μm. **(C, D)** Western blot analysis of the lysates from sphere-forming SAS cells, collected at the time points indicated in A. Expression levels of the canonical Hippo pathway–related proteins (C) and the LATS1/2 axis–related proteins and phosphorylations (D) were examined by probing with the indicated antibodies. Arrow, arrowhead, and asterisk indicate MST2-pT180, LATS2-pS835, and non-specific bands, respectively. α-tubulin was used as a loading control. A indicates SAS-a (attached SAS cells under adherent culture conditions). The ratio of band intensity was shown in Supplementary Table 1.

To examine the roles of LATS1/2 in the initiation of sphere formation, we analyzed the temporal expression patterns and activation states of LATS1/2 during sphere formation by SAS cells. The cells were transferred onto an ultra–low attachment surface plate in sphere culture medium without serum, and then incubated for 1 h to 10 days (Figure 4A). Sphere morphology was monitored by observing time-dependent changes in spheres formed by single cells. Each cell divided into two daughter cells after approximately 1 day (24 h) of culture under sphere-forming conditions, and subsequently grew, generating a small floating sphere at 3–5 days and a large typical sphere with round smooth contours at 7–10 days (Figure 4B). Western blot analyses revealed that levels of LATS1/2 were markedly elevated at 6–24 and 12–24 h, respectively, but decreased or disappeared at 48 and 72 h (Figure 4C, top and second panels; Supplementary Table 1). Moreover, LATS1/2 were strongly phosphorylated and activated by MST kinase(s), with a peak at 12 h. Notably, phosphorylation of LATS2 on T1041 was temporarily reduced to undetectable levels after transfer of cells into sphere medium, and was maintained at low levels for 6 h, but then increased dramatically at 12 h (Figure 4C, fourth panel from top). Unlike LATS2, phosphorylation of LATS1 on T1079 was maintained at low levels, similar to those in adherent SAS cells, until 6 h (Figure 4C, third panel from top). Consistent with these results, MST1/2 (pT183/pT180) were phosphorylated and activated with a peak at 3–6 h, whereas TAZ levels (phosphorylated and unphosphorylated forms) were increased and stabilized in response to inactivation of LATS2 at 1–6 h (Figure 4C, sixth and seventh panels from top). However, no increase in the YAP level was observed during sphere formation (Figure 4C, bottom panel). Based on these results, we categorize the process of sphere formation into four stages, as follows: (1) early stage (1–6 h); (2) pre–self-renewal (pre-SR) stage, a preliminary phase of self-renewal (12 h); (3) initiation of self-renewal (in-SR) stage, in which self-renewal (cell division) is executed (24 h); and (4) sphere formation stage, during which mature spheres grow (from about 48 h onward after initiation of the culture under sphere-forming conditions) (Figure 4A). In this context, it is likely that the completion of sphere formation requires the following steps: activation of TAZ, mediated by downregulation of LATS2, during the early stage; transient re-activation of LATS1/2 during the pre-SR and in-SR stages, which is accompanied by degradation of TAZ; and finally inactivation of LATS1/2 during the sphere formation stage. Our results also suggest that constitutive activation or inhibition of LATS or TAZ throughout sphere formation fails to confer self-renewal capacity on CSCs (Figures 2, 3).

Generation of mammalian cells with CSC properties involves the EMT and its regulators, e.g., SNAIL and TWIST [6]. Moreover, the EMT promotes self-renewal through the Hippo pathway; specifically, downregulation of Scribble and the subsequent activation of TAZ promote breast cancer stemness [21]. Because SNAIL activity is correlated with LATS2-mediated T203 phosphorylation during the EMT [25], we asked whether SNAIL is phosphorylated by LATS2 during sphere formation by SAS cells. As expected, T203 of SNAIL became intensely phosphorylated after incubation under sphere-forming conditions. After 6–24 h, when LATS1/2 were upregulated, the protein appeared as multiple bands, likely due to other phosphorylation events by unknown kinases (Figure 4D, top panel, lanes 4–6). Consistent with this, expression of N-cadherin, a mesenchymal marker and transcriptional target of SNAIL, was induced at 24–72 h immediately after the activation of LATS and SNAIL, suggesting that self-renewal of OSCCs is also associated with the EMT (Figure 4D, third panel from top). Interestingly, phosphorylation of T203 on SNAIL diminished at 48–72 h, similarly to LATS (Figure 4D, top panel, lanes 7 and 8), but accumulation of TAZ was no longer restored by a reduction in LATS activity (Figure 4C, seventh panel from top, lanes 7 and 8).

Cell cycle and DNA damage checkpoint regulators are also thought to play important roles in promoting CSC-like properties, including sphere formation, radioresistance, EMT, and tumorigenesis [20, 49]. Indeed, the DNA damage checkpoint kinases CHK1 and CHK2 are highly expressed and activated in CSCs of gliomas, in which they promote efficient DNA repair, thereby inducing radioresistance [50]. Moreover, the mitotic kinase Aurora-A (AURKA) is also highly expressed in the nucleus of breast CSCs, and promotes sphere formation, radioresistance, invasion, and metastasis in breast and laryngeal cancer cells [51, 52, 53]. Therefore, these proteins represent potential cancer stemness markers. CHK1/2 and Aurora-A directly phosphorylate and activate LATS2 via a Hippo–YAP/TAZ axis–independent pathway [46, 54, 55]. Therefore, we examined the expression patterns of CHK1/2 and Aurora-A during sphere formation of SAS cells. The expression levels of these kinases were significantly elevated and maintained during the pre-SR and in-SR stages (12–24 h), and then modestly decreased during the sphere formation stage (Figure 4D, fourth to sixth panels from top, lanes 5–8), suggesting that these kinases also play important roles in self-renewal of CSCs in OSCC and other cancer cell lines. Moreover, the expression levels of CHK1 and Aurora-A and the phosphorylation level of S408 on LATS2 by CHK1 were higher in adherent SAS cells than in non-CSCs such as HSC3 and HSC4 (Supplementary Figure 1C, fourth to seventh panels). Interestingly, the phosphorylation level of S835 on LATS2 by CHK1-dependent LATS2 trans-autophosphorylation, which is important for LATS2 activation [55], was increased during sphere formation and peaked at 12–24 h, whereas S408 phosphorylation level was maintained at comparatively low levels during the early stage and gradually decreased after the early stage (Figure 4D, ninth and tenth panels from top). The dephosphorylation of S408 after the early stage might also play an important role in sphere formation. Notably, c-MYC, a transcription factor that functions as an oncoprotein, is highly expressed in nasopharyngeal carcinoma cells and transcriptionally activates CHK1 and CHK2 in CSCs [56]. Consistent with this, c-MYC was highly expressed during the early stage of sphere formation by SAS cells, prior to the increases in CHK1 and CHK2 levels (Figure 4D, eighth panel from top, lanes 2–4). Expression of Scribble was maintained at comparatively high levels during the early stage and decreased slightly after the pre-SR stage (from 24 h onward), but did not dramatically oscillate between stages of sphere formation like LATS and TAZ (Figures 4D, seventh panel; 2A, fourth panel).

Taken together, these results suggest that phosphorylation and activation of LATS1/2 and SNAIL are required for initiation of self-renewal (i.e., the acquisition of self-renewal capacity) during sphere formation by CSCs, whereas dephosphorylation and inactivation of these proteins might be required for the maintenance of sphere formation during later stages. Notably in this regard, the expression levels and activities of LATS1/2, SNAIL, and TAZ are collaboratively up- or downregulated at the appropriate stages (timing) during sphere formation, which may be essential for self-renewal of CSCs.

### SNAIL, CHK1, and Aurora-A are required for sphere formation in CSCs of OSCC

**Figure 5:**
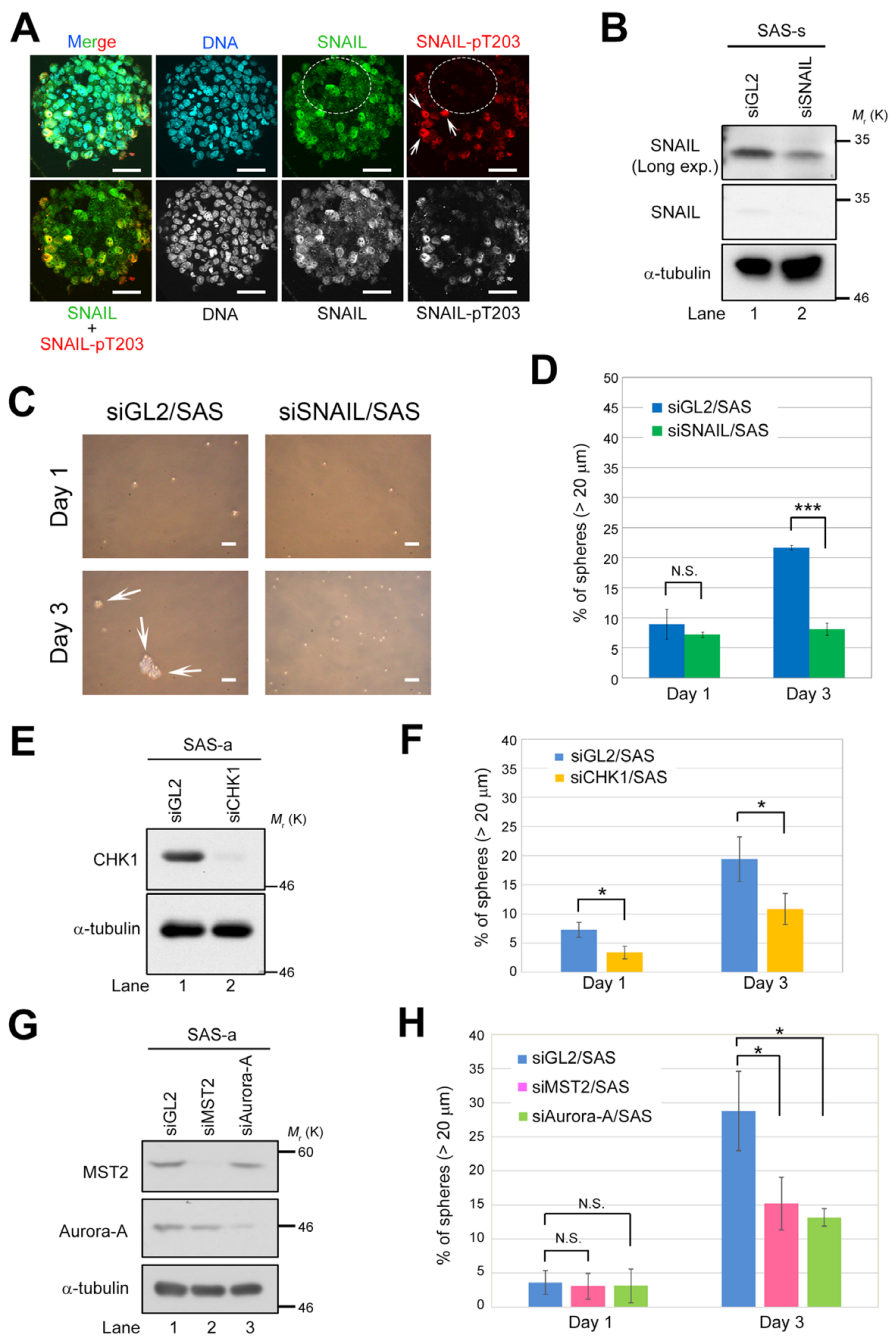
Deficiency of LATS2 axis–related proteins prevents sphere formation by SAS cells **(A)** Immunofluorescence staining of SAS sphere with anti-SNAIL and anti–SNAIL-pT203 antibodies. Nuclei were counterstained with Hoechst 33258. White arrows indicate cells positive for SNAIL-pT203 expression. Dashed circles show cells negative for SNAIL-pT203 expression (red) but positive for SNAIL expression (green). Scale bars, 50 μm. **(B)** Western blot analysis of SNAIL in sphere lysates from control (siGL2) and SNAIL-knockdown (siSNAIL) SAS cells. α-tubulin was used as a loading control. **(C)** Representative phase contrast images of SNAIL-depleted (siSNAIL/SAS) and control SAS (siGL2/SAS) cells, grown under sphere formation conditions, on days 1 and 3. White arrows indicate spheres. Scale bars represent 100 μm. **(D)** Frequency of sphere formation in siSNAIL/SAS (green bars) and siGL2/SAS (blue bars) cells, grown under sphere formation conditions, on days 1 and 3. More than 120 cells were counted on day 1, and more than 140 cells were counted on day 3. **(E)** Western blot analysis of CHK1 in lysates from control (siGL2) and CHK1-knockdown (siCHK1) SAS cells grown under adherent culture conditions. α-tubulin was used as a loading control. **(F)** Frequency of sphere formation in siCHK1/SAS (yellow bars) and siGL2/SAS (blue bars) cells, grown under sphere formation conditions, on days 1 and 3. More than 120 cells were counted. **(G)** Western blot analysis of MST2 and Aurora-A in lysates from control (siGL2), MST2-knockdown (siMST2), and Aurora-A–knockdown (siAurora-A) SAS cells grown under adherent culture conditions. α-tubulin was used as a loading control. **(H)** Frequency of sphere formation in siMST2/SAS (pink bars), siAurora-A/SAS (light green bars), and siGL2/SAS (blue bars) cells, grown under sphere formation conditions, on days 1 and 3. More than 150 cells were counted on day 1, and more than 50 cells were counted on day 3.

To further validate the importance of the LATS2–SNAIL axis in self-renewal, we fluorescently immunostained mature spheres of SAS cells with anti–SNAIL-pT203 (a LATS2 phosphorylation site) and anti-SNAIL antibodies (Figure 5A). Cells positive and negative for SNAIL-pT203 expression were mixed in mature spheres, and expression of SNAIL protein was not necessarily correlated with cellular location within the sphere (e.g., inner vs. outer cells). Moreover, nuclear localization of SNAIL-pT203 was also observed in the staining-positive cells (Figure 5A, white arrows), consistent with a previous observation that LATS2-mediated phosphorylation of T203 promotes nuclear accumulation and stabilization of SNAIL protein during EMT progression [25]. These results suggest that a mature sphere consists of heterogeneous cell populations that exhibit diverse molecular signals at different stages of sphere formation: pT203-positive cells may reflect CSCs vigorously undergoing self-renewal, whereas negative cells may reflect CSCs in which self-renewal was somewhat limited during sphere formation (Figure 5A, white arrows and dashed open circles).

**Figure 6:**
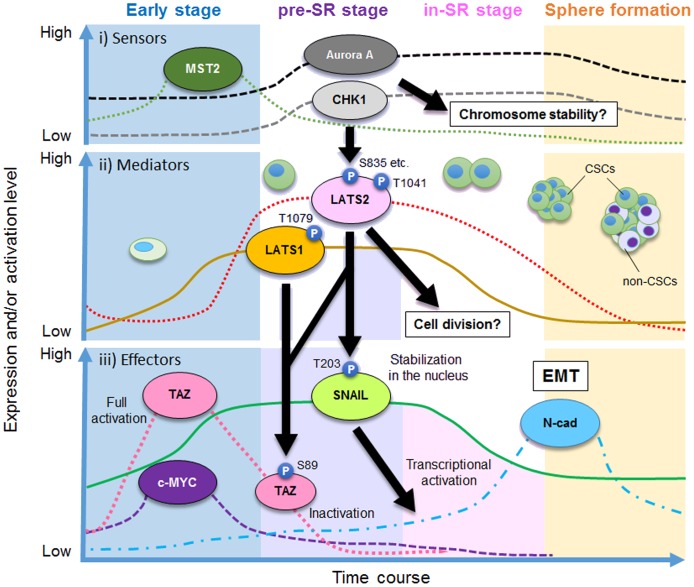
Model of the role of LATS1/2 kinases in the pre–self-renewal (pre-SR) stage during sphere formation by CSCs The process of sphere formation of CSCs consists of four stages: early, pre-SR, initiation of self-renewal (in-SR), and mature sphere formation. LATS1/2 are required for self-renewal via regulation of TAZ and SNAIL. The solid and dashed lines show imaginary lines, not actual values, of independent expression and/or activity levels of the indicated proteins. Black arrows show the direction of the signaling cascade found in this study.

To confirm that SNAIL is essential for sphere formation in CSCs of OSCC, we transfected SAS cells with siRNA against SNAIL (siSNAIL/SAS), cultured the transfectants for 48 h, and then performed sphere formation assays in the absence of serum. Knockdown of SNAIL successfully inhibited sphere formation by SAS cells, even though SNAIL was not completely depleted (as determined by western blotting), suggesting that a threshold level of SNAIL is critical for sphere formation (Figure 5B–5D). Because T203 phosphorylation by LATS2 stabilizes SNAIL protein in the nucleus, these results also suggest that the LATS2–SNAIL axis promotes self-renewal. Similarly, knockdown of CHK1, MST2, or Aurora-A also successfully inhibited sphere formation (Figure 5E–5H), indicating that, like SNAIL and LATS1/2, these proteins are essential for self-renewal of CSCs of OSCC. Because the upstream factors of LATS, including MST2, CHK1, and Aurora-A, are required for sphere formation, the MST2/CHK1/Aurora-A–LATS1/2 axis might contribute to self-renewal of CSCs in OSCC through phosphorylation of SNAIL and/or TAZ (Figure 6).

## DISCUSSION

In this study, we demonstrated that LATS1/2 are essential for self-renewal of OSCC cells with CSC properties, probably via activation of SNAIL and TAZ. Because we previously reported that LATS1/2 are pivotal in ensuring that cell division generates two daughter cells harboring equal genetic material [29, 57, 58, 59], the putative self-renewal initiation proteins, including LATS1/2, CHK1/2, and Aurora-A, are expressed at the highest levels during the process of sphere formation, and are likely activated to appropriately initiate and promote the successful completion of symmetric (i.e., self-renewing) cell division (Figure 6).

In mammalian germ line stem cells, Aurora-A promotes symmetric division by regulating spindle orientation via PLK1 and the scaffold protein Gravin/AKAP12 on the mother centrosome [60], and maintains self-renewal and pluripotency in embryonic stem cells by inhibiting p53-directed expression of differentiation-associated genes [61]. Notably, overexpression of Aurora-A promotes self-renewal of breast CSCs by stabilizing Wnt3a mRNA via suppression of miR-128, which collaborates with SNAIL [62]. Consistent with this, SNAIL positively regulates expansion and activity of breast CSCs via binding and subsequent degradation of p53 [63], and controls the switch between self-renewal and differentiation in skeletal stem cells via an interaction with YAP/TAZ [64]. Moreover, in colorectal CSCs, SNAIL can also promote symmetric self-renewing division by inhibiting Numb expression via miR-146a [65]. These results suggest that SNAIL is a key factor not only in regulating the EMT, but also in promoting self-renewing symmetric division of CSCs. Importantly, LATS2 phosphorylates and stabilizes SNAIL during the EMT [25], which might contribute to initiation of self-renewal and symmetric division of CSCs (Figures 2, 4 and 5).

In mouse embryonic stem cells, LATS2 sustains stemness with pluripotency by inhibiting epigenetic repression of *Oct4* and *Nanog*, probably by interacting with PRC2 to promote histone H3-K27 trimethylation, and can also execute the differentiation program in a p53-dependent manner [66, 67]. Although LATS2 stabilizes p53 in human cancer cells by inhibiting MDM2 [68], it also might maintain stemness of SAS cells by inhibition of p53-dependent differentiation; SAS cells harbor a mutant p53 protein in which both the tetramerization domain and the C-terminal regulatory domain are deleted due to a truncation at E366 (http://p53.free.fr/Database/Cancer_cell_lines/p53_cell_lines.html). However, because these deleted regions of p53 are dispensable for the interaction between p53 and SNAIL [63], the LATS2–SNAIL axis could ensure full execution of self-renewal in SAS cells via SNAIL-mediated suppression of p53. By contrast, in non-CSC OSCC cell lines, although HSC3 cells lack any detectable p53 and HSC4 cells highly express a mutant p53 (R248Q) [69], LATS1/2 were expressed at relatively low levels in these cell lines (Figure 1).

At the early stage of sphere formation before the pre-SR stage, expression and activity of LATS2 were transiently decreased in a Scribble-independent manner, which may be important for the early stage–specific accumulation of TAZ (Figure 4). Because OCT4 and NANOG repress the expression of LATS2 by binding to a near region of the *LATS2* gene [30, 70], these proteins (which are highly expressed in SAS cells [43]) might be responsible for transient suppression of LATS2 during the early stage. TAZ confers self-renewal capacity on non-CSC breast cancer cells, acting downstream of the induction of EMT and subsequent inactivation of Scribble [21]. Furthermore, expression of *LATS1/2* is downregulated by promoter methylation in developing OSCC tissues [71], which are probably heterogeneous cell populations containing mostly non-CSCs. Therefore, the transient suppression of LATS2 during the early stage may be essential for acquisition of CSC properties during the earlier phase prior to initiation of self-renewing cell division. However, we found that, in SAS cells, TAZ was activated before the EMT (SNAIL activation and N-cadherin induction) in a Scribble-independent manner, suggesting that the molecular mechanisms by which non-CSCs acquire self-renewal capacity differ slightly between OSCC and breast cancer.

Collectively, our results provide a new insight into the molecular mechanisms by which CSCs execute self-renewal and non-CSCs acquire the property of stemness. This knowledge should facilitate the development of advanced medical treatments targeting CSCs expressing LATS kinases.

## MATERIALS AND METHODS

### Cell culture

Three OSCC cell lines, HSC3, HSC4, and SAS, were maintained in Dulbecco's modified Eagle's medium (DMEM, Sigma-Aldrich, St. Louis, MO, USA) supplemented with 10% fetal bovine serum (FBS, Hyclone, Logan, UT, USA), 100 U/mL penicillin, and 100 μg/mL streptomycin, and incubated at 37°C and 5% CO_2_. These cell lines were provided by the RIKEN Bio Resource Center, Ibaraki, Japan [44]. SCC-4 cells were obtained from the JCRB Cell Bank (#JCRB9118; Osaka, Japan) and were cultured in DMEM/F12K (1:1) supplemented with 10% FBS, 100 U/mL penicillin, 100 μg/mL streptomycin, and 0.4 μg/mL hydrocortisone (Sigma-Aldrich).

### Sphere formation assay

Cells were trypsinized and mechanically dissociated by repeating shearing through a 25G injection needle to avoid a cell aggregation (i.e., doublets and triplets). Single cells were plated in EZ-Bind Shut II (IWAKI, Chiba, Japan) at 400 cells/cm^2^ and grown in sphere culture medium [DMEM/F-12K (1:1), supplemented with B27 (Invitrogen, San Diego, CA, USA), 20 ng/mL EGF (Sigma-Aldrich), 20 ng/mL bFGF (Sigma-Aldrich), 2 mM glutamine (Invitrogen), and penicillin/streptomycin]. After incubation at 37°C and 5% CO_2_ for the indicated times, the spheres were analyzed by microscopy. To examine earlier stages of self-renewal, spheres with diameters of larger than 20 mm were counted.

### Subcellular fractionation

Cells were separated into cytoplasm, cell membrane, nucleus, and cytoskeleton fractions using the ProteoExtract Subcellular Proteome Extraction Kit (S-PEK; Calbiochem, La Jolla, CA, USA). Prior to electrophoresis, the proteins were denatured by boiling for 7 min in Laemmli sample buffer.

### Treatment with MG132

SAS cells (2 × 10^5^ cells/dish) were plated in 60 mm dishes. Five days after seeding, the cells were exposed to 20 μM MG132 (Calbiochem) in DMEM/10% FBS medium and incubated for 2 h at 37°C under 5% CO_2_. After washing in PBS (-), the cells were fractionated by S-PEK.

### Western blot analysis

To prepare the total cell extracts, the cells were lysed by RIPA buffer (20 mM Tris-HCl, pH 7.5, 150 mM NaCl, 1% Triton X-100, 0.1% SDS, 1% sodium deoxycholate) supplemented with 100 μg/mL PMSF, 1 μg/mL aprotinin, 10 μg/mL leupeptin, 1 μg/mL pepstatin A, 1 mM NaF, 1 mM Na_3_VO_4_, 10 mM β-glycerophosphate. The denatured proteins were separated by SDS-PAGE and transferred onto PVDF membranes (Immobilon P; Millipore, Billerica, MA, USA). After blocking with 5% skim milk or 5% BSA in TBST (20 mM Tris-HCl, pH 7.5, 150 mM NaCl, 0.05% Tween 20), the membranes were probed with the indicated primary antibodies, followed by washing in TBST and incubation with HRP-conjugated secondary antibodies. Protein bands were visualized using the Western Lightning Plus ECL reagent (Perkin Elmer, Waltham, MA, USA). The intensities of bands were quantified using ImageJ software.Full scan images of blots were shown in Supplementary Figure 3.

### Antibodies

Commercial antibodies were obtained from the indicated suppliers: Lamin A/C (4C11), LATS1 (C66B5), LATS1-pT1079 (D53D3), SNAIL (L70G2) monoclonal and E-cadherin, MAPK, MST2, N-cadherin, p-MST1/2 (pT183/pT180), and YAP-pS127 polyclonal antibodies from Cell Signaling Technology (Danvers, MA, USA); vimentin (VIM 3B4) monoclonal antibody from American Research Products (Waltham, MA, USA); YAP (H-9) monoclonal and 14-3-3γ and Scribble polyclonal antibodies from Santa Cruz Biotechnology (Dallas, TX, USA); 14-3-3γ (pS58) polyclonal antibody from Abcam (Cambridge, UK); actin (AC-40), α-tubulin (B-5-1-2), CHK1 (DCS-310), and CHK2 (DCS-270) monoclonal and TAZ (WWTR1) polyclonal antibodies from Sigma-Aldrich; LATS2 polyclonal antibody from Bethyl Laboratories (Montgomery, TX, USA); Myc (9E10) and Myc (PL14) monoclonal antibodies from Upstate (Bedford, MA, USA) and MBL (Nagoya, Japan), respectively; and GAPDH monoclonal antibody from Fitzgerald (Acton, MA, USA). Aurora-A, LATS2-pS408, LATS2-pS835, LATS2-pT1041, SNAIL-pT203, and YAP (GS) polyclonal antibodies were described previously [25, 46, 54, 55, 58].

### Plasmids, siRNAs, and transfection

Human YAP2 ORF was isolated by PCR-based cloning from a human myometrium cDNA library. The full-length human YAP2 cDNA was inserted into the *Asc*I and *Not*I sites of mammalian expression vector pCMV6myc (+AscI), a modified version of pcDNA3. Plasmid DNAs were transfected into SAS cells using Lipofectamine and PLUS reagents (Invitrogen). At 48 h after transfection, the cells were trypsinized and subjected to the sphere formation assay. For western blot analysis, lysates were prepared from cells cultured for 1 day under sphere formation conditions (SAS-s).

Sequences of siRNA duplexes were as follows: siLATS1 (#3509), 5’-ACUUUGCCGAGGACCCGAAdTdT-3’; siLATS2 (#31), 5’-CCGCAAAGGGUACACUCAACUCUGUdTdT-3’; siTAZ (#2), 5’-AGAGGUACUUCCUCAAUCAdTdT-3’; siCHK1 (#477), 5’-UCGUGAGCGUUUGUUGAACdTdT-3’; and siGL2 (firefly luciferase), 5’-CGUACGCGGAAUACUUCGAdTdT-3’. siSNAIL (Human SNAI1, ##SR304489C) and siMST2 (Human STK3, #SR304635B) were purchased from OriGene Technologies (Rockville, MD, USA). siRNA duplexes were introduced into HSC3, SAS, or SCC-4 cells using Oligofectamine or Lipofectamine 2000 (Invitrogen). At 48 h after transfection, the cells were trypsinized and subjected to the sphere formation assay. For western blot analysis, lysates were prepared from the cells cultured for 1 day (Figures 2A, 2C, 5B and Supplementary Figure 2A) or 3 days (Supplementary Figure 1D) under sphere formation conditions (HSC3-s or SAS-s) or conventional adherent culture conditions (HSC3-a, SAS-a, or SCC-4-a).

### Immunofluorescence

Spheres were plated on cover glasses in 6-well plates and fixed with 3.5% paraformaldehyde in PBS (-) for 10 min at room temperature. The sphere cells were then permeabilized with 0.2% Triton X-100 in PBS (-) for 5 min at room temperature. For indirect immunofluorescence (IF), the cells were blocked with 2% BSA in PBS (-) for 1 h, incubated overnight at 4°C with anti-SNAIL-pT203 polyclonal and anti-SNAIL monoclonal antibodies, and then incubated with secondary antibody (Alexa Fluor 488 goat anti-rabbit IgG or Alexa Fluor 594 goat anti-mouse IgG; Molecular Probes, Eugene, OR, USA) for 1 h at room temperature. After the samples were washed with PBS (-), DNA was counterstained with Hoechst 33258 (SIGMA). The samples were treated with SlowFade reagent (Thermo Fisher Scientific, Waltham, MA, USA) and observed on a FV10i FLUOVIEW confocal laser scanning microscope (Olympus, Tokyo, Japan).

### Statistical analysis

Statistical analysis was performed in Microsoft Excel. All data on sphere formation assays are shown as means and standard deviations of three independent experiments, except for those in Figure 3E, 3F and Supplementary Figure 2B (n = 1). *P*-values were calculated using Student's t-test. ^*^p < 0.05, ^**^p < 0.01, and ^***^p < 0.001. N.S., no significant difference.

## SUPPLEMENTARY MATERIALS FIGURES AND TABLES


