# Supplementary material for: LATS1/2 kinases trigger self-renewal of cancer stem cells in aggressive oral cancer
Source: Oncotarget. 2019 Feb 1;10(10):1014–30. doi: 10.18632/oncotarget.26583 (PMC6383686; doi:10.18632/oncotarget.26583)
Supplement: Supplementary file 1 [file oncotarget-10-1014-s001.pdf]

LATS1/2 kinases trigger self-renewal of cancer stem cells in aggressive oral cancer

SUPPLEMENTARY MATERIALS

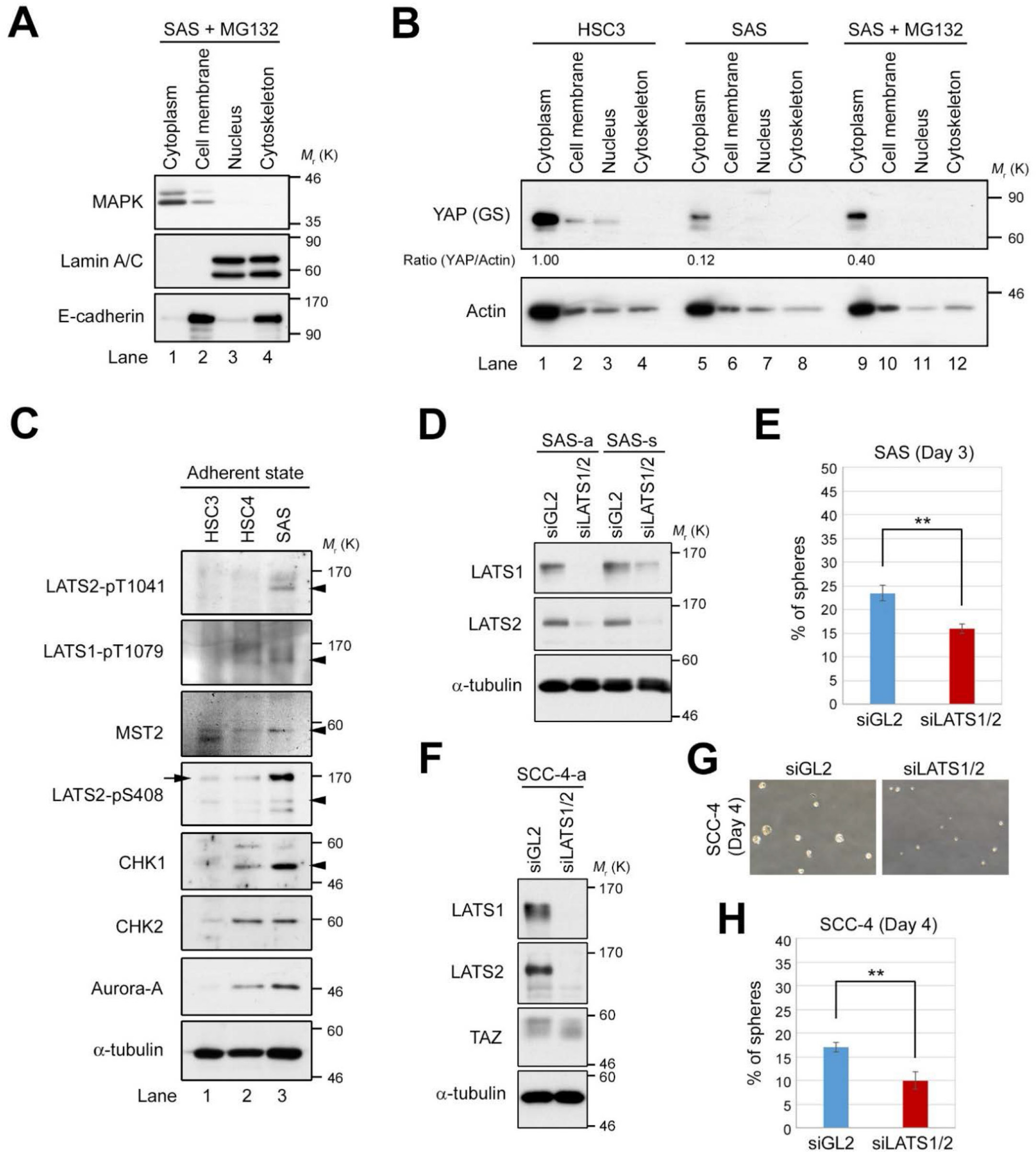

Supplementary Figure 1: LATS1/2 are moderately activated and YAP is degraded in adherent SAS cells. (A) Western blot analysis of subcellular fractionated lysates from MG132-treated SAS cells (20 µM, 2 h), using the indicated antibodies as fraction markers. **(B)** Western blot analysis of YAP in subcellular fractionated lysates from HSC3 cells (as a control), non-treated SAS, and MG132-treated SAS cells. The level of cytoplasmic YAP was normalized against that of cytoplasmic actin. **(C)** Western blot analysis of LATS1/2 axis–related proteins in HSC3, HSC4, and SAS cells grown under adherent culture conditions. Arrowheads show the predicted band sizes of the indicated proteins. Arrow shows putative hyper-phosphorylating shifted bands from LATS2-pS408. α-tubulin was used as a loading control. **(D, F)** Western blot analysis of LATS1 and LATS2 in LATS1/2-double knockdown SAS-a, SAS-s (D), and adherent SCC-4-a (F) cells. **(E, H)** Frequency of sphere formation by SAS (E) and SCC-4 (H) cells transfected with siLATS1/2 and siGL2. More than 600 cells were counted on day 3 or 4. **(G)** Representative phase contrast images of LATS1/2-depleted (siLATS1/2) and control SCC-4 (siGL2) cells, grown under sphere formation conditions, on day 4.

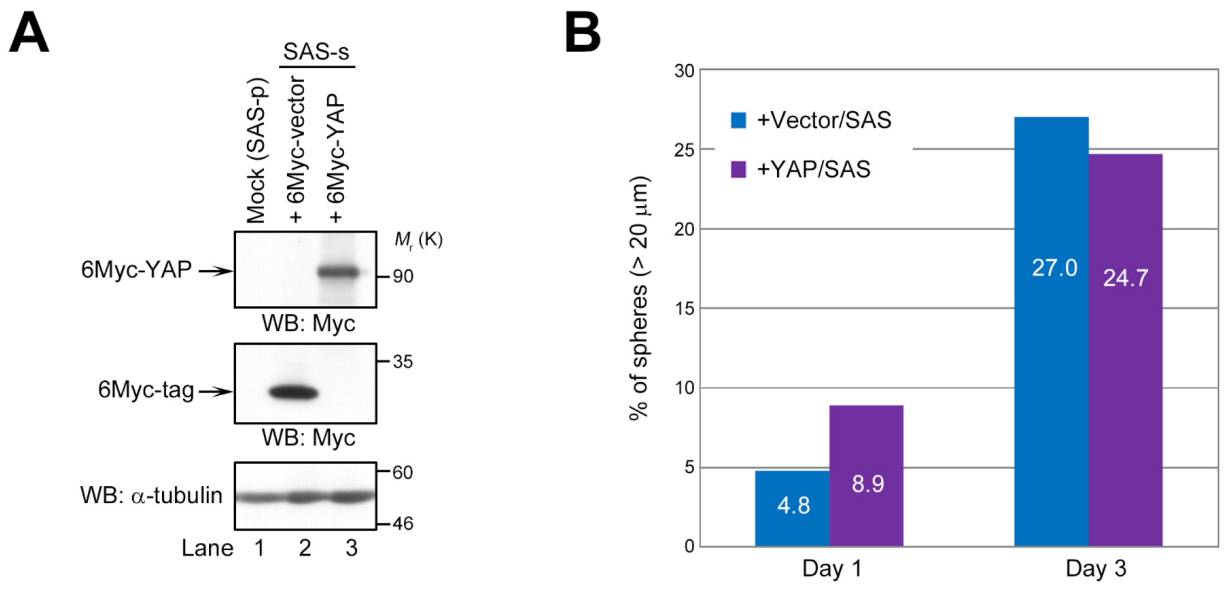

Supplementary Figure 2: Enforced overexpression of YAP neither inhibits nor promotes sphere formation by SAS cells. (A) SAS cells were transfected with 6Myc-tagged YAP or 6Myc-vector alone, cultured for 48 h, and then additionally cultured for 24 h under sphere formation conditions (SAS-s). "Mock" indicates non-transfected SAS cells cultured under conventional adherent conditions (SAS-a). YAP expression was monitored by western blot with anti-Myc antibody. α-tubulin was used as a loading control. **(B)** Frequency of sphere formation in SAS cells expressing 6Myc-YAP (+YAP/SAS, purple bars) or vector alone (+Vector/SAS, blue bars), grown under sphere formation conditions, on days 1 and 3. The numbers in the bar graphs show percentages of sphere formation. More than 150 cells, including spheres, were counted in each experiment.

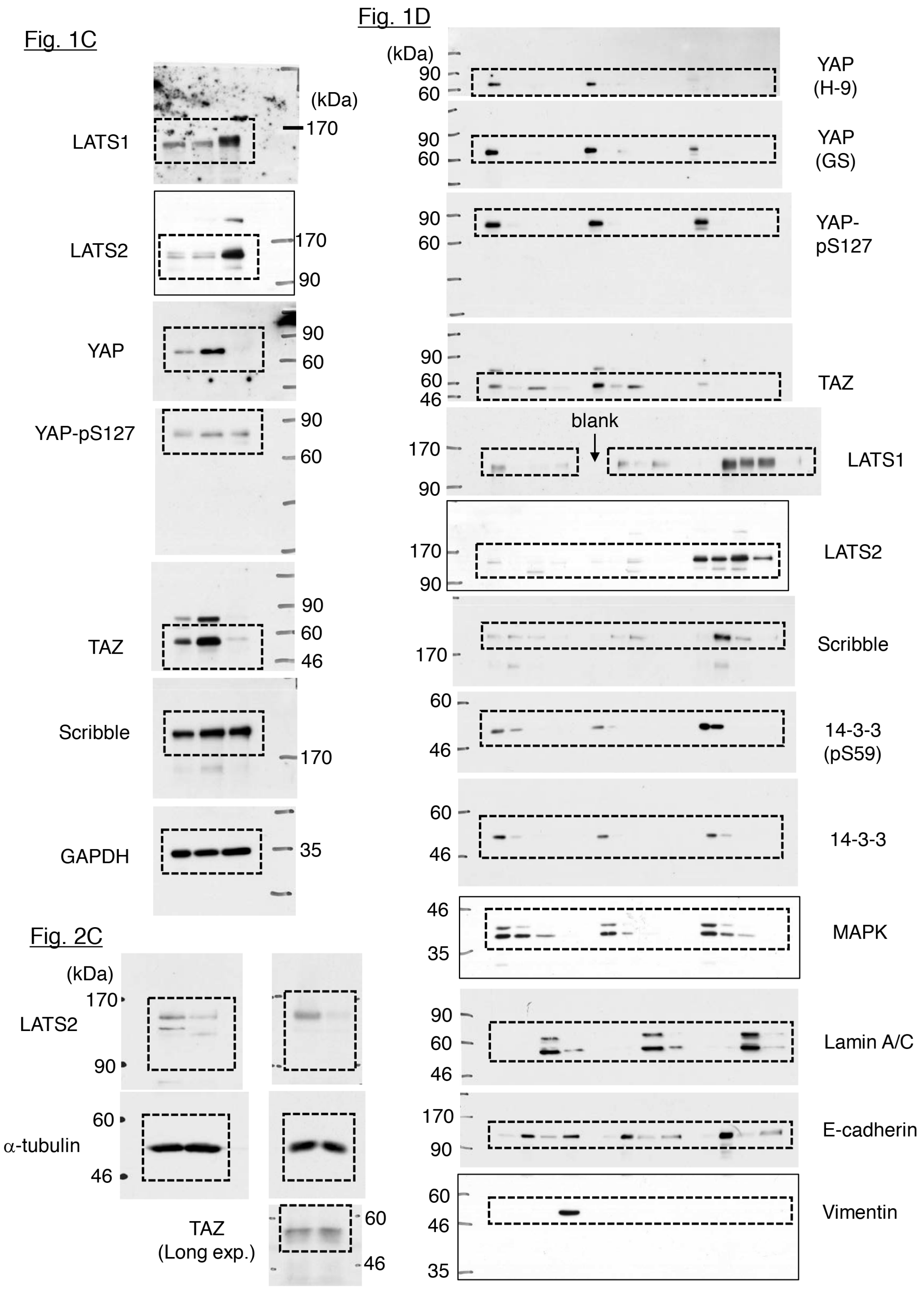

Supplementary Figure 3: Full scan images of Figures 1C, 1D, and 2C.

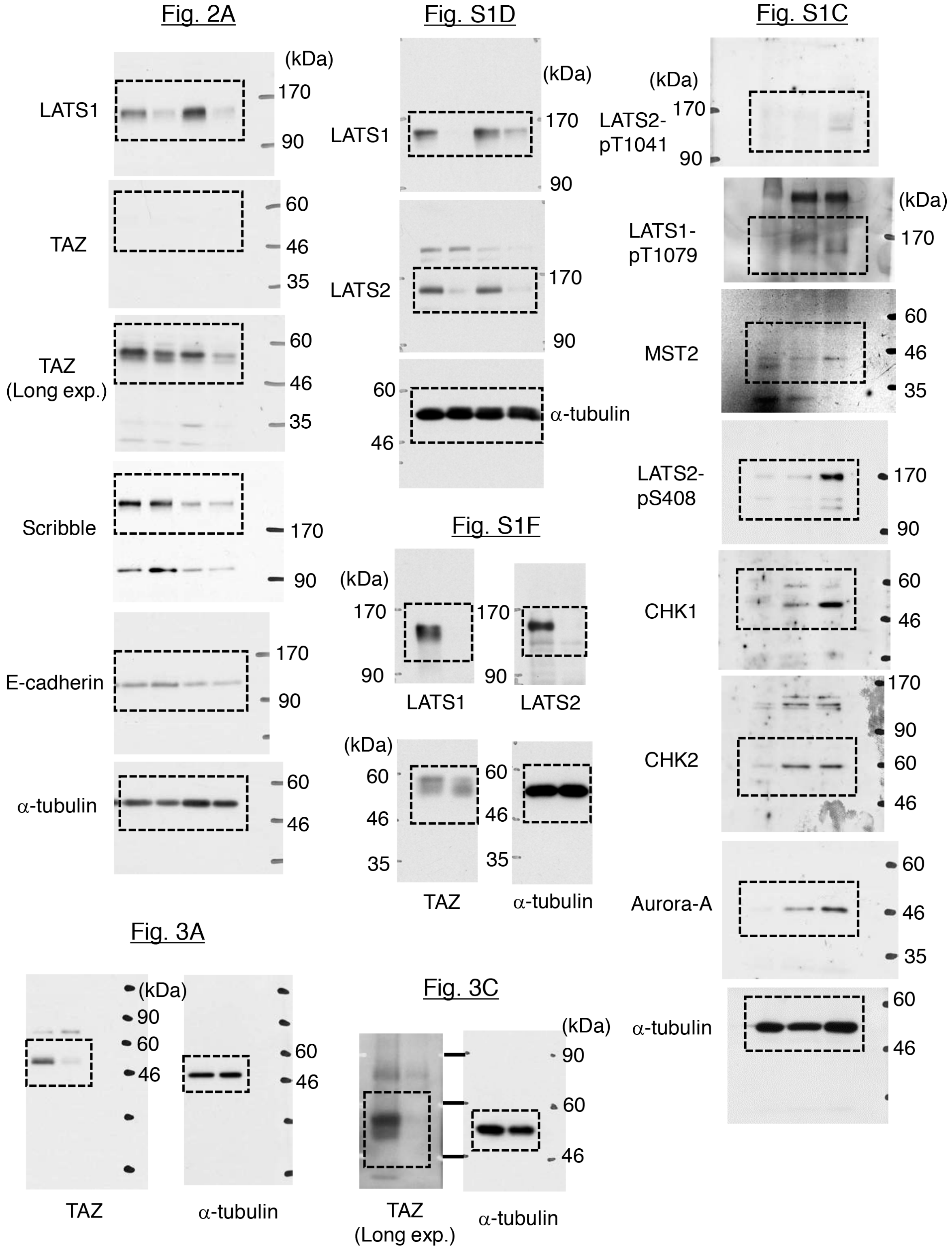

Supplementary Figure 3: Full scan images of Figures 2A, 3A, 3C, S1C, S1D, and S1F.

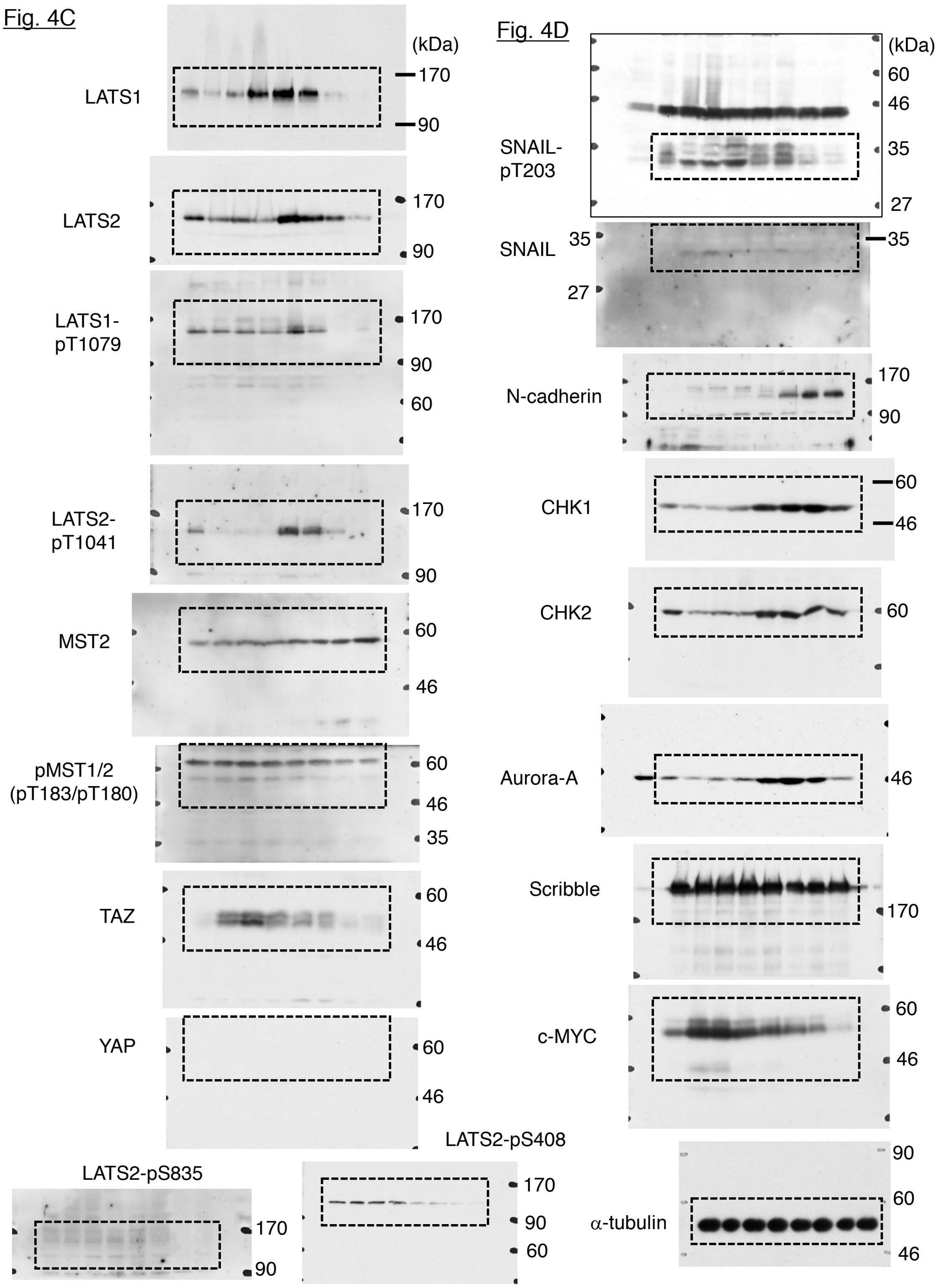

Supplementary Figure 3: Full scan images of Figure 4C and 4D.

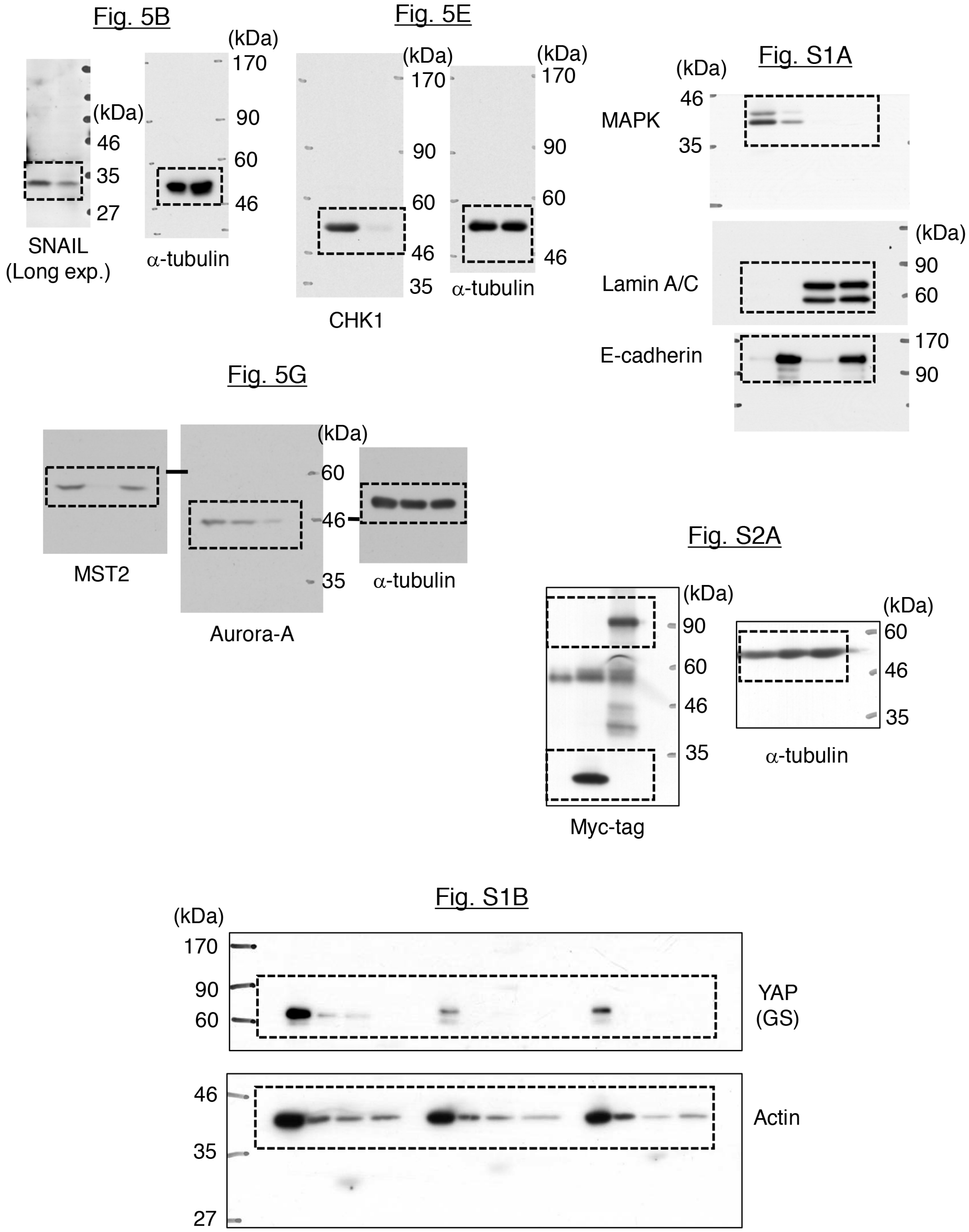

Supplementary Figure 3: Full scan images of Figures 5B, 5E, 5G, S1A, S1B, and S2A.

Supplementary Table 1: The ratio of band intensity at the indicated time during sphere formation to that at adherent culture in Figure 4C and 4D

Proteins	Adherent (A)	Sphere formation						
		**1 h**	**3 h**	**6 h**	**12 h**	**24 h**	**48 h**	**72 h**
LATS1	1.0	0.5	1.0	4.2	6.8	3.7	0.3	0.0
LATS2	1.0	0.9	1.0	0.7	3.3	1.8	1.2	0.3
LATS1-pT1079	1.0	1.2	1.2	1.2	2.4	0.8	0.0	0.2
LATS2-pT1041	1.0	0.1	0.1	0.1	5.1	3.6	0.8	0.0
MST2	1.0	2.1	1.9	1.8	1.6	1.0	1.5	2.5
p-MST1/2	1.0	1.4	1.2	1.0	0.8	0.6	0.3	0.2
TAZ	1.0	19.7	24.8	14.3	10.2	7.5	1.8	1.8
SNAIL-pT203<br>(Upper bands)	1.0<br>(1.0)	1.1<br>(0.8)	1.3<br>(1.2)	2.7<br>(3.6)	1.7<br>(2.6)	2.3<br>(4.0)	0.7<br>(0.8)	0.2<br>(0.3)
CHK1	1.0	0.6	0.3	1.6	6.1	7.8	12.2	3.3
CHK2	1.0	0.5	0.7	0.7	2.5	2.3	2.2	1.4
Aurora-A	1.0	0.6	0.8	1.0	2.4	3.5	3.2	1.4
Scribble	1.0	1.5	1.2	1.4	1.3	0.7	1.1	1.0
c-Myc	1.0	5.7	4.1	3.4	2.7	2.0	1.4	0.3
LATS2-pS835	1.0	1.4	1.2	1.5	2.0	1.3	0.1	0.1
LATS2-pS408	1.0	1.5	1.2	1.1	0.3	0.2	0.1	0.0

The levels of the indicated proteins were normalized against the level of α-tubulin.
